# Mad, bad and dangerous to know: the biochemistry, ecology and evolution of slow loris venom

**DOI:** 10.1186/1678-9199-19-21

**Published:** 2013-09-27

**Authors:** K Anne-Isola Nekaris, Richard S Moore, E Johanna Rode, Bryan G Fry

**Affiliations:** 1 Nocturnal Primate Research Group, Oxford Brookes University, Oxford OX3 0BP, UK; 2 International Animal Rescue, Ciapus, Bogor, Indonesia; 3 Venom Evolution Lab, School of Biological Sciences, University of Queensland, St. Lucia, Queensland 4072, Australia

**Keywords:** Venoms, Ecology, Primates, Intraspecific competition, Predation, Ectoparasite, *Naja naja*

## Abstract

Only seven types of mammals are known to be venomous, including slow lorises (*Nycticebus* spp*.*). Despite the evolutionary significance of this unique adaptation amongst *Nycticebus,* the structure and function of slow loris venom is only just beginning to be understood. Here we review what is known about the chemical structure of slow loris venom. Research on a handful of captive samples from three of eight slow loris species reveals that the protein within slow loris venom resembles the disulphide-bridged heterodimeric structure of Fel-d1, more commonly known as cat allergen. In a comparison of *N. pygmaeus* and *N. coucang,* 212 and 68 compounds were found, respectively. Venom is activated by combining the oil from the brachial arm gland with saliva, and can cause death in small mammals and anaphylactic shock and death in humans. We examine four hypotheses for the function of slow loris venom. The least evidence is found for the hypothesis that loris venom evolved to kill prey. Although the venom’s primary function in nature seems to be as a defense against parasites and conspecifics, it may also serve to thwart olfactory-orientated predators. Combined with numerous other serpentine features of slow lorises, including extra vertebra in the spine leading to snake-like movement, serpentine aggressive vocalisations, a long dark dorsal stripe and the venom itself, we propose that venom may have evolved to mimic cobras (*Naja* sp.). During the Miocene when both slow lorises and cobras migrated throughout Southeast Asia, the evolution of venom may have been an adaptive strategy against predators used by slow lorises as a form of Müllerian mimicry with spectacled cobras.

## Introduction

The study of the venomous systems of animals, including invertebrates, snakes, lizards and frogs, has provided remarkable insight into their interactions with predators, prey and competitors, as well as yielding promising medical advances through development of pharmacological agents [1]. Offensive and defensive venom systems in mammals are far rarer and are comparatively little known. Of the species known or suspected to be venomous, virtually nothing is known about Haitian solenodons (*Solenodon paradoxurus*); studies of the venom of European water shrews (*Neomys fodiens*) and American short-tailed shrews (*Blarina brevicauda*) are restricted mainly to capture-recapture studies; researchers still cannot resolve if European hedgehogs (*Erinaceus europaeus*) are truly venomous [2,3]; recent detailed research on the platypus (*Ornithorhynchus anatinus*) reveals strong convergence between reptile and mammal venomous systems [4]; the oral secretions of vampire bats have only recently been intensively studied, revealing a suite of complex venomous proteins [5]. Whittington *et al*. [4] point out that the study of chemical and genetic aspects of venom can help to elucidate the evolution of this rare trait in mammals. Dufton [6] posits that our knowledge of mammal venom is only in its infancy, and that even more species of mammals may harbour venomous adaptations.

**Figure 1:**
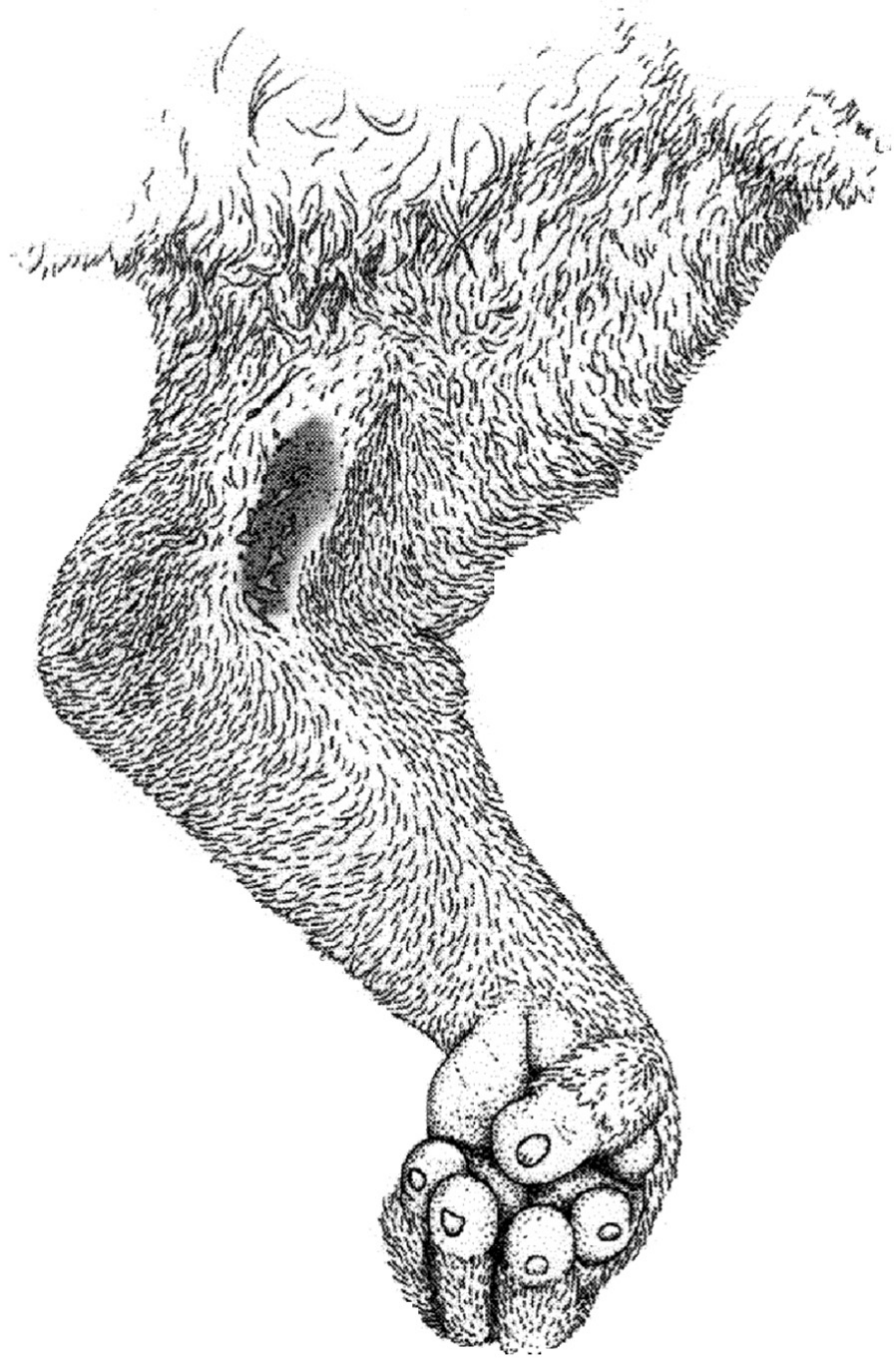
The slow loris brachial gland (dark oblong area on the inside of the elbow region).

**Figure 2:**
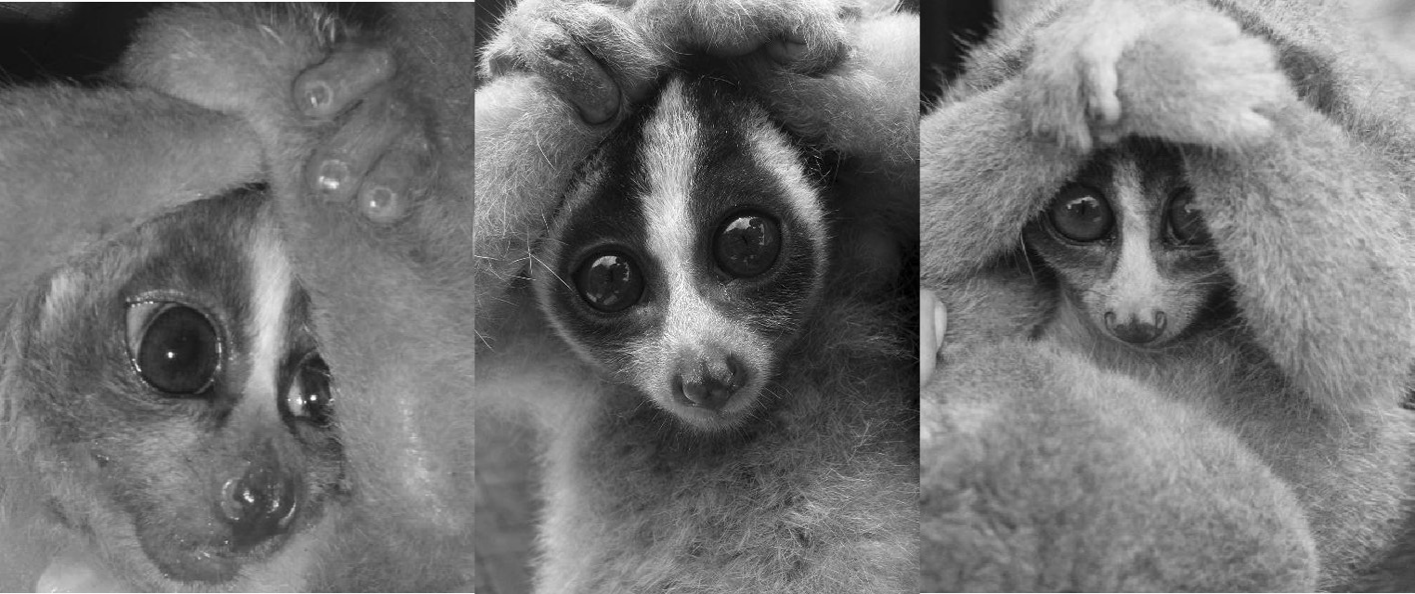
**Slow lorises in defensive posture, whereby the arms are raised above the head to combine saliva with brachial gland exudate: *N. menagensis*, *N. javanicus *and *N. coucang*.**

The slow lorises of Southeast Asia (*Nycticebus* spp*.*) are the final mammals, and the only primates, which harbour toxins. The venom is usually delivered after a threatened loris raises it arms above its head, combining fluid of its brachial gland (Figure 1) with saliva [7]. In this classic defence posture (Figure 2), the mouth can quickly be moved to the brachial gland to combine the fluids, and the mixture applied to the top of the head for defence or kept in the mouth to bite [8]. Alterman [8] also demonstrated that the slow loris’ procumbent anterior incisors, or toothcomb, normally ascribed feeding and grooming functions, are effective as a venom delivery system by conducting liquid upward. Despite the animals’ small size (~300 g – 2 kg), slow loris bites are intensely painful, and in both humans and loris conspecifics can cause oedema, fester, take weeks to heal, and leave loss of fur and scarring. In extreme cases, bite recipients may enter anaphylactic shock, sometimes resulting in death [9-11] (Research report to the Nocturnal Primate Research Group, Oxford Brookes University). Despite the extraordinary nature of this adaptation within a primate, its function and chemistry still remains little known. In this review, we detail current knowledge of the ecology and biochemistry of loris venom, and provide current data for the most probable hypotheses regarding its function. Any research carried out by the authors was approved by the Oxford Brookes subcommittee for ethics in animal research and followed guidelines set out by the Animal Behaviour Society.

## Review

Dufton [6] suggested that local folklore may be the best starting point for uncovering new venomous taxa. Adopting such an approach, Dumbacher *et al*. [12] characterized potent defensive toxins in the skin and feathers of New Guinean birds – the pitohuis. Delving further into anthropological knowledge, Dumbacher *et al*. [13] also identified batrachotoxins in the New Guinean blue-capped ifrita and in melyrid beetles, which are consumed by the birds and may be the source of the toxin. Similarly, folklore in Thailand, Laos, Myanmar, Indonesia, Cambodia, China and Vietnam can be traced back centuries, revealing tales of the loris’ bad taste and toxic bite; although intriguing, until now, these stories have been collected on an *ad hoc* basis [10,14].

Nekaris [15] and Nijman and Nekaris [16] systematically collected such folktales in Java, where beliefs in the toxicity of slow lorises varied across six regencies. The slow loris’ bite was widely regarded to be dangerous or fatal in four of the five regencies visited. Knowledge of loris venom extends to Indonesian pet traders who routinely cut out the lorises’ front teeth to prevent their biting potential purchasers [17]. In Sukabumi Regency, the removed teeth were also believed to possess black magic properties. Respondents in five of six regencies linked lethal potency of lorises to their blood. For example, in one community in Sukabumi Regency, residents recount that before their ancestors went to war, they smeared their swords in loris blood. When they pierced their enemies with the anointed weapons, the wounds would fester, and death would follow. In Tasikmalaya and Garut Regencies, residents described how if a drop of blood or semen touched the ground, a landslide would follow, whereas in Sukabumi Regency, if the placenta of a loris touched the ground, nothing could ever grow there again. In Sumedang and Ciamis Regencies, however, few myths prevailed and lorises were considered economically valuable and suitable for hunting.

Local beliefs only give us a starting point to search for the nature and function of slow loris venom. Alterman [8] was the first researcher to show with *in vivo* experiments that loris venom can actually kill other animals. In a set of experiments where he injected secretions from captive greater slow lorises (*N. coucang*) into mice, Alterman discovered that loris venom was only deadly when secretions from a loris’ brachial gland (brachial gland exudates – BGE) were combined with saliva. Death rates of mice differed based on the extract used to dissolve the toxin. From this Alterman concluded that lorises may possess two toxin types: one fast-acting aqueous toxin and a second toxin that enters the circulatory system more slowly, although he was not able to characterise this biochemically.

Krane *et al.*[18] extracted BGE without saliva from a single old zoo-housed individual – probably *N. bengalensis.* Of several methods used, high performance liquid chromatography (HPLC) was the most effective at identifying organic compounds in the sample. In particular, they found that protein in the BGE shared a high degree of sequence similarity with the disulphide-bridged heterodimeric structure of Fel-d1, more commonly known as cat allergen. They interpreted the propensity of only some individuals to suffer anaphylaxis from loris bites as consistent with variable sensitivity to a protein allergen.

Following this, Hagey *et al.*[7] studied eight captive pygmy slow lorises *N. pygmaeus* and eight captive greater slow lorises *N. coucang.* When examined by Hagey *et al.*[7] using gas chromatography/mass spectrometry (GC/MS), brachial gland exudates contained a complex mixture of volatile and semi-volatile compounds. They observed 212 different compounds in *N. pygmaeus*, identifying a wide variety of aromatic compounds consistent with dietary absorption from a species maintained on a captive frugivorous diet, and a concurrent difficulty in complete metabolism of this chemical class of compounds. The remaining identified compounds were a series of C4-C7 aldehydes, ketones, and acetates. They found 68 different compounds present in *N. coucang*, 33 (48%) of which were unique to the species. To examine the exudate oil contents by a different approach, samples from both loris species were examined by nano-electrospray ionization mass spectrometry (nano-ESI-MS). Although the sugars glucose, neuraminic acid, and a variety of fatty acids (fa) were detected, none were present in amounts sufficient to constitute the exudate oil itself. Notably absent from the profile were phospholipids.

**Figure 3:**
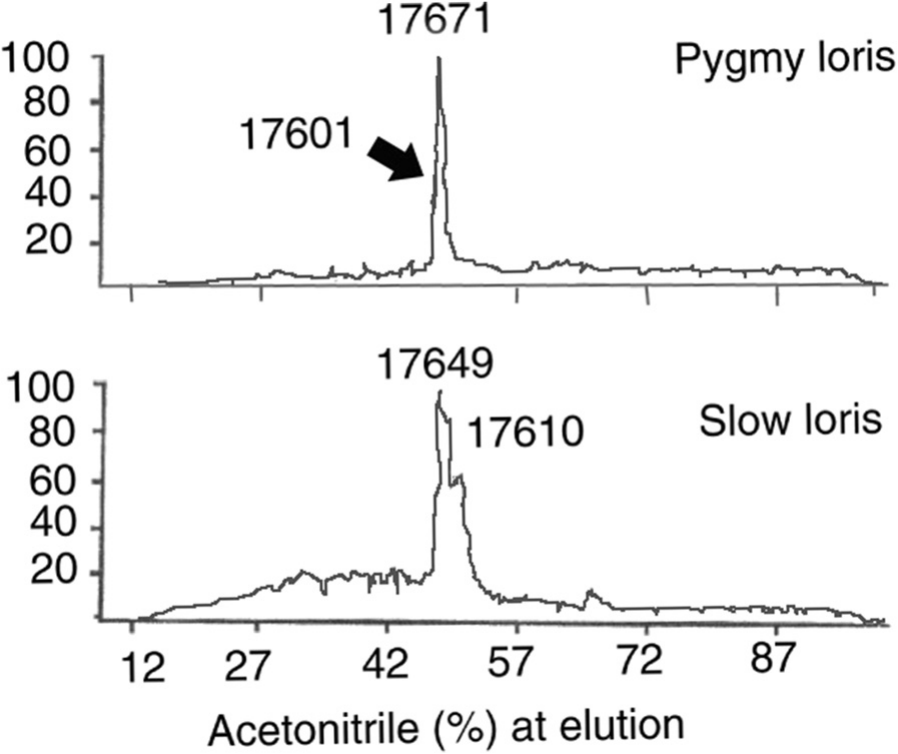
Comparison of pygmy and greater slow loris LC/MS profiles and 4 A and B sequence alignment.

**Figure 4:**
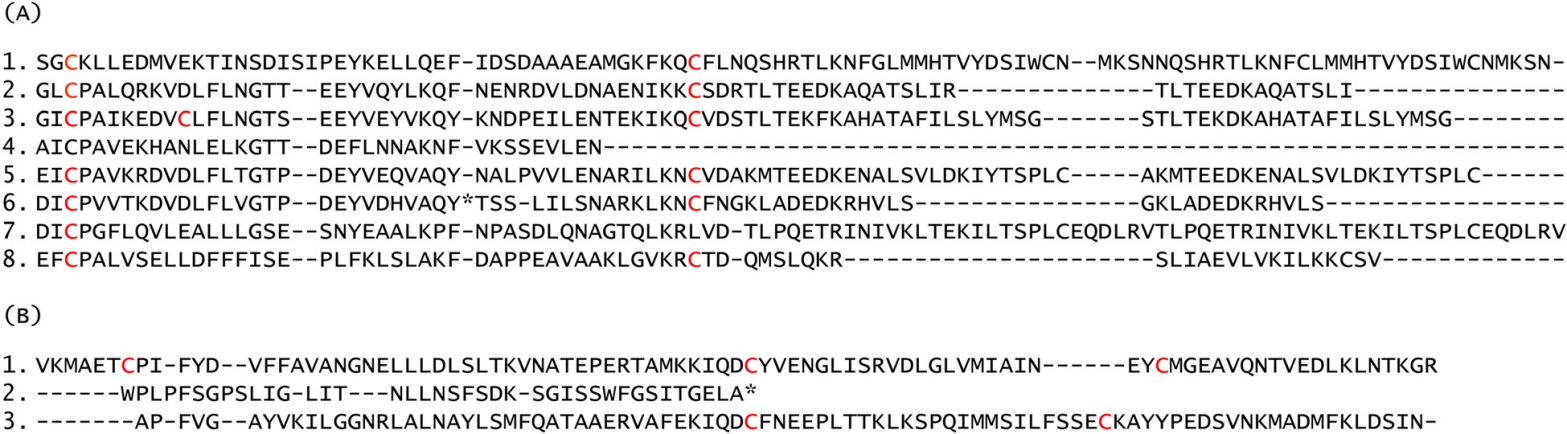
**NH2-terminal amino acid sequences of the pygmy loris α- and β-chains that make up the 18k major peptide of brachial gland exudate. (A)** Comparison between the pygmy loris α-chain sequence and members from each clade of the α-chain superfamily: 1. secretoglobin (3288868); 2. mouse salivary androgen binding protein (19919338); 3. mouse putative protein 20948528; 4. loris brachial gland secretion; 5. domestic cat allergen; 6. human genome putative protein; 7. uteroglobin (6981694); and 8. lipophilin (5729909). Numbers refer to NCBI accession numbers. Homologous amino acids are highlighted in grey. **(B)** Comparison between the pygmy loris β-chain sequence and two members with similar β-chains. 1. domestic cat allergen (423192); 2. loris brachial gland secretion β-chain; and 3. mouse salivary protein (19353044).

Liquid chromatography/mass spectrometry (LC/MS) analysis of the brachial gland secretion from both species also revealed that each contained a single dominant protein component, molecular weight 17.6 k (Figure 3). Both taxa contained two isoforms (*N. pygmaeus* – 17671 and 17601 daltons; *N. coucang* – 17649 and 17610 daltons). Reduction of the disulfide bonds in the 17.6k peptide revealed that it was a heterodimer of two smaller peptides, molecular weights 7.8 kDa (α-chain) and 9.8 kDa (β-chain) linked together by two disulfide bridges. Sequencing of the α/β-chains showed that the loris brachial gland peptide is a new member of the secretoglobin (uteroglobin/Clara cell 10k) family. As found by Krane *et al.*[18], loris peptide was assigned to subfamily 4, with a close sequence homology with domestic cat Fel-d1 chain I peptide [19,20] (Figure 4 A and – B). The secretoglobin family is characterized by small lipophilic peptides found as major constituents in a variety of mammalian secretions. These proteins are all α/β-homo- and heterodimers stabilized by two or three intramolecular cystine disulfide bonds. In what is termed the uteroglobin-fold, the α- and β- monomers are formed from grouping four α-helices, and (for the two monomers) the combined eight α-helix bundle folds to form a pocket for the binding of different hydrophobic molecules [21].

This simple structural motif of the uteroglobin fold stands in sharp contrast to the wide array of biological activities assigned to this group of proteins. In the loris 17.6k protein, Hagey *et al.*[7] hypothesised that the smaller α-chain may form a slightly pyramidal-shaped lid that is hinged along one edge by the two disulfide bridges to the larger β-chain, forming a unit roughly in the shape of a cigar box. The α-subunit may have a shallow hydrophobic centre in the lid, which sits over a similar but deeper pocket in the β-chain box, forming a molecular snare for a small hydrophobic molecule. They based this hypothetical potential, as similar molecular docking of hydrophobic molecules like progesterone, polychlorinated biphenyls and retinol has been shown using the crystal structure of human uteroglobin [22]. Other than the disulfide bridges located together in the “hinge”, only these interacting hydrophobic regions hold the lid to the box. When the snare is in the hydrophobic environment of the secreted oil, the lid is free to open and the box can accept a signaling molecule. One function for the box would be to hold a species-specific message, and the varying compositions of the α/β chains in different species support this idea [23]. Cats heavily contaminate their environments with Fel-d1, using the protein not as a toxic defence, but as a species recognition molecule [24]. What evidence is there that loris toxin has a function in nature or in experimental settings? Does it seem to be more than just a communication device? We review four potential hypotheses that have been explored to various degrees.

### Prey as target

Slow lorises are fauni-frugivores that consume a wide variety of animal prey and plant materials, including those that contain considerable amounts of secondary metabolites [25,26]. They easily overcome relatively large prey including birds, bats, lizards and tarsiers; venom could be useful in subduing these animals. Many animals use *de novo* or acquired toxins to subdue prey, broadly classed in two types: excitatory and depressant [27]. Excitatory toxins induce sustained contraction paralysis, whereas depressant toxins induce a slow and flaccid paralysis. Short-tailed shrews use depressant toxins to immobilise prey, which they cache for later consumption [28]. Excitatory toxins are useful for predators that may need to release their prey, and should be less likely in an arboreal environment where immobilised prey may fall to the forest floor [29].

Until now, we have found no evidence to support this hypothesis. Although loris venom can kill small prey, venom is not used to paralyse it, and we know of no instance of lorises caching prey [8]. Of hundreds of field hours and observations of prey capture events, lorises consume prey rapidly and effectively with powerful jaws and sharp teeth, and it does not seem that venom is required to carry this out. This does not rule out the possibility that they sequester secondary compounds from certain foods.

### Predators as target

Alterman [8] held that loris venom was almost certainly a defence against predators, and that loris venom is used to anoint adults and young against predators. A single observation of a Javan slow loris (*N. javanicus*) mother heavily covering her offspring with venom before leaving it for a few hours provides the only support for this hypothesis so far from the field, during a 16-month study of the ecology of slow loris venom (Nekaris, pers. obs.). Slow lorises have classically been described to avoid predators by crypsis [30]. Morphological specialisations of loris postcranial anatomy allow them to remain still until a potential threat has passed. Thickened nuchal skin may serve as a last-minute defence if a predator does strike. Forbey *et al.*[31] predicted that less mobile animals that cannot readily flee from predators will be more likely to exploit highly-toxic secondary metabolites as camouflage. Loris mobility is 'reduced’ beyond morphological constraints in two ways. Lorises go through a period of seasonal torpor during periods of food scarcity [10]; travelling and social behaviour substantially decrease, with lorises sleeping alone rather than in small groups. Lorises also 'park’ their young from the age of 6 weeks. Locomotor dexterity is undeveloped; they cannot escape rapidly, their ability to allogroom is limited, and their immunological parasite-defence machinery is not yet fully developed. Chewing material and licking or rubbing it on to fur has been observed in many mammals [32,33]. Through this mechanism, chemically-defended mothers may pass defences to their offspring [34,35]. Both *de novo* and acquired toxins can serve to make prey unpalatable, meaning that they survive encounters with predators and escape unharmed [36]. This sequestration of toxins may also serve as a form of camouflage. Many animals ingest secondary metabolites and accumulate them in their tissues, including pitohuis (*Pitohui* sp.) that sequester batrachotoxins from melyrid beetles (Cleroidea) [12]. That such toxins may not have been detected in captive lorises is not surprising – even the highly-noxious dendrobatid poison-dart frogs do not contain detectable amounts of toxin when raised in captivity [13,37].

Evidence from both the lab and the field suggest that loris venom may repel some predators. Alterman [8] presented BGE+saliva to potential predators. He found that loris secretions repelled cats (*Panthera pardis, Panthera tigris, Neofelis nebulosa*), sun bears (*Helarctos malayanus*), and civets (*Paradoxurus hemaphroditus, Arctictis binturong*). Nekaris (unpublished data) reconstructed this experiment with sun bears (n = 2) and Bornean orangutans (*Pongo pygmaeus,* n = 2). Whereas both bears not only rapidly retreated from swabs permeated with loris BGE, but also began stereotypic pacing, both orangutans (known loris predators, Hardus *et al*. [38]) consumed the swabs and the related foliage. In all cases, the reactions were in less than a minute showing the effectiveness, or lack thereof, of the scent.

If loris venom is effective against olfactory-orientated predators, we would expect that few animals would be lost to such predators [39]. During three long-term field studies using radio-tracking, no loris has been known to be lost to a nocturnal mammalian predator [26,40-42]. Indeed, lorises have been observed to walk within meters of civets and small leopard cats, even when carrying young, with seeming ambivalence ([30] and Nekaris and Rode, personal observation).

### Ectoparasites as target

In mammals, fur is the first line of defence against consumers, and may serve as a repository for chemicals [43]. In social animals like primates, grooming typically serves a key function for reducing parasites [44]. For lorises, however, during solitary torpor and infant parking, anointment with a secondary compound could provide an essential line of defence, and can also protect areas of the body where a loris cannot groom itself [45]. In birds, many factors influence ectoparasite reduction; nest composition may be modified by adding leaves with antiparasitic properties, or shape of the nest may be altered, influencing internal temperature [31,46]. Lacking nests, anointing infants directly would provide a powerful chemical alternative for slow lorises.

Prevalence and intensity of ectoparasite infestation among the Lorisidae is extremely low compared to other primates [47]. While eight of nine wild studies of six taxa revealed no or few ectoparasites (*Loris tardigradus*, *L. lydekkerianus lydekkerianus*, *L. l. nordicus*, *Nycticebus pygmaeus*, *N. bengalensis*, *N. javanicus*), only one study of *N. coucang* conducted during the wet season found a small amount of ticks in all animals [48]. In a preliminary experiment to test the potency of loris venom on ectoparasites, Nekaris (unpublished data) used loris BGE+saliva diluted three times with purified water and applied this solution with a cotton swab to 12 individual leeches. Leeches were collected in the village and weighed approximately 0.03 g each. All leeches died upon coming into contact with the solution (range 128 sec – 480 sec; mean = 265 sec ± 104.4).

### Conspecifics as target

The loris brachial gland may mirror the defensive spur of the male platypus, which has evolved as a seasonal offensive weapon used only during the breeding season, and could explain why loris venom is only sometimes potent to its recipients [4]. Alternatively, the venom could be used for intersexual competition. Male lorises have large testes, which could be a sign of high male-male competition for females. The few days during which mating can occur are replete with intense competition and fighting between males and females. Throughout the year, females maintain tightly defended territories that they share only with their offspring and one to three other males [15]. Bite wounds in captivity and in the wild have been a major cause of morbidity and mortality, with fatal head wounds being the most common [26,49]. In a review of 30 years of morbidity records from North American zoos, Fuller *et al.*[50] found that trauma was a significant contributor of mortality to both adult and immature animals. Indeed, several animals died following bite wounds that were chronically non-healing, leading to necrosis, septicaemia, lung edema, and cellulitis. These non-healing wounds are also frequent in rescue centres, with multiple veterinarians stating that if a loris bites another, its chances of surviving are slim [10].

It is possible that venom is costly to produce and lorises may only activate it when they need it. In one of two recorded cases of a human entering anaphylactic shock after a loris bite [9], the loris delivering the bite had previously nipped his owner several times. It was only when the loris had been introduced to a conspecific with which it fought, and the owner separated the two, that a potentially deadly bite was delivered, causing the owner to go into anaphylaxis.

**Figure 5:**
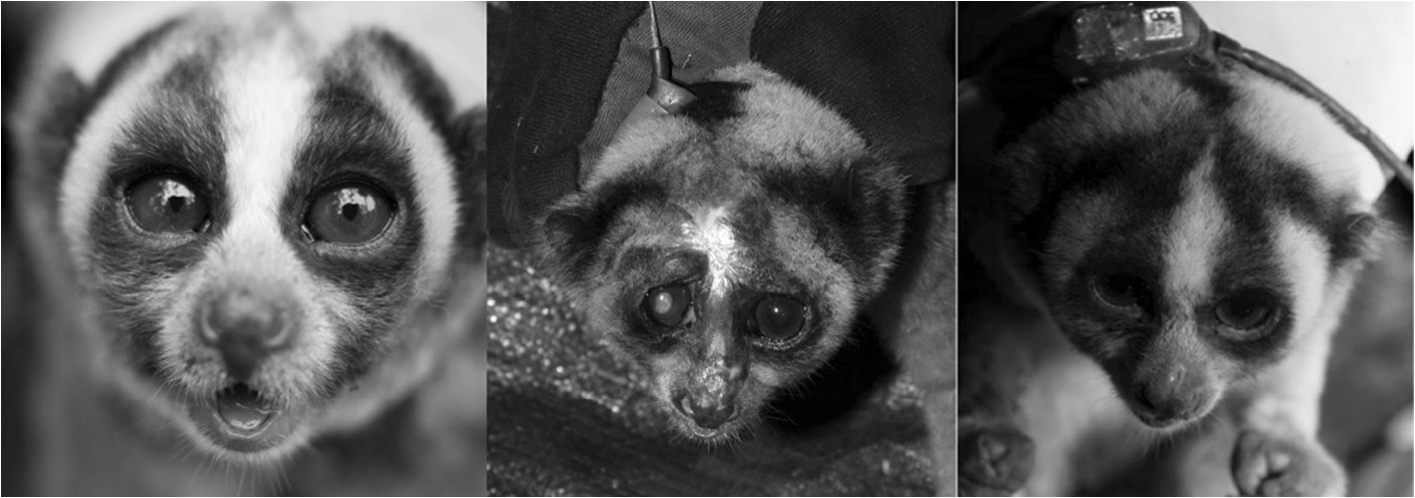
**Male wild *Nycticebus javanicus*, from Cipaganti near Garut, Java, during three successive captures in April 2012, November 2012 and February 2013, showing his appearance before receiving a severe conspecific bite wound, just afterwards, and 3 months afterwards.**

Wounds and scars are often observed in animals sold on wildlife markets (EJR and KAIN, personal observation). They also are reported by loris hunters, who reject wild animals with wounds, as they are not as profitable [15]. In a study of wild *N. javanicus* conducted by KAIN and EJR in West Java, during the study time of 16 months, 13 of 28 animals (46%) were found to have wounds, scars or broken/missing/stiff digits. There was no difference between sexes in adult and sub-adult animals (χ^2^ = 3.1, df = 1, p = 0.543, n = 25). All four adult males had high relative testes volume (> 3.5 mm^2^) and had wounds, suggesting possible intraspecific competition. In 29 captured wild *Nycticebus coucang* 52% of males and 12% of females had fresh or old wounds; the difference was significant [48]. In five cases where the authors observed relatively fresh wounds or scars in wild *N. javanicus*, injuries healed completely by the next capture. These included two animals with very severe and potentially lethal injuries: one head wound of a female loris resembled a complete removal of half of the head scalp including the ear; the other injury of a male adult and its development are shown in Figure 5. Many of these wounds may originate during mating, as during the 18-months in Java, agonistic events were rare, but always occurred during mating. Between bouts of fighting, male lorises have been observed to lick their brachial gland and anoint themselves heavily, presumably with toxin (Additional file 1). Perhaps the toxin began as a warning signal [7], but over time, evolved into a true venom against other lorises, with the instant reaction of a loris to cover its head from an agonistic conspecific, as a reaction to protect its most vulnerable body part.

## Evolution of loris venom

Field and laboratory studies are still ongoing as researchers attempt to understand the function and ecological role of loris venom. Furthermore, upcoming analyses of the first samples of venom from wild lorises may address some of the gaps presented here. But what event in the evolutionary history of slow lorises might have driven the venom selection? Deception provides the basis for mimicry in nature. Many animals possess protective colouration that deceives predators by masquerading as something else [51,52]. Mimicry is common among insects [53], with many caterpillars (Lepidopteran larvae) displaying extremely convincing imitations of various species of snake, both in appearance and behaviour [54]. Mimicry among vertebrates is less common and in mammals extremely rare [55]. Across the natural world imperfect mimicry is widespread [56]. In order to gain protection, a mimic need not perfectly replicate its model, as long as it is similar enough to cast uncertainty in the mind of the predator [55,57]. This replication may be aposematic, olfactory, auditory or a combination of the three with the ultimate goal to cast uncertainty into the mind of a both generalist and specialist predators, as well as remaining cryptic to its own prey.

**Figure 6:**
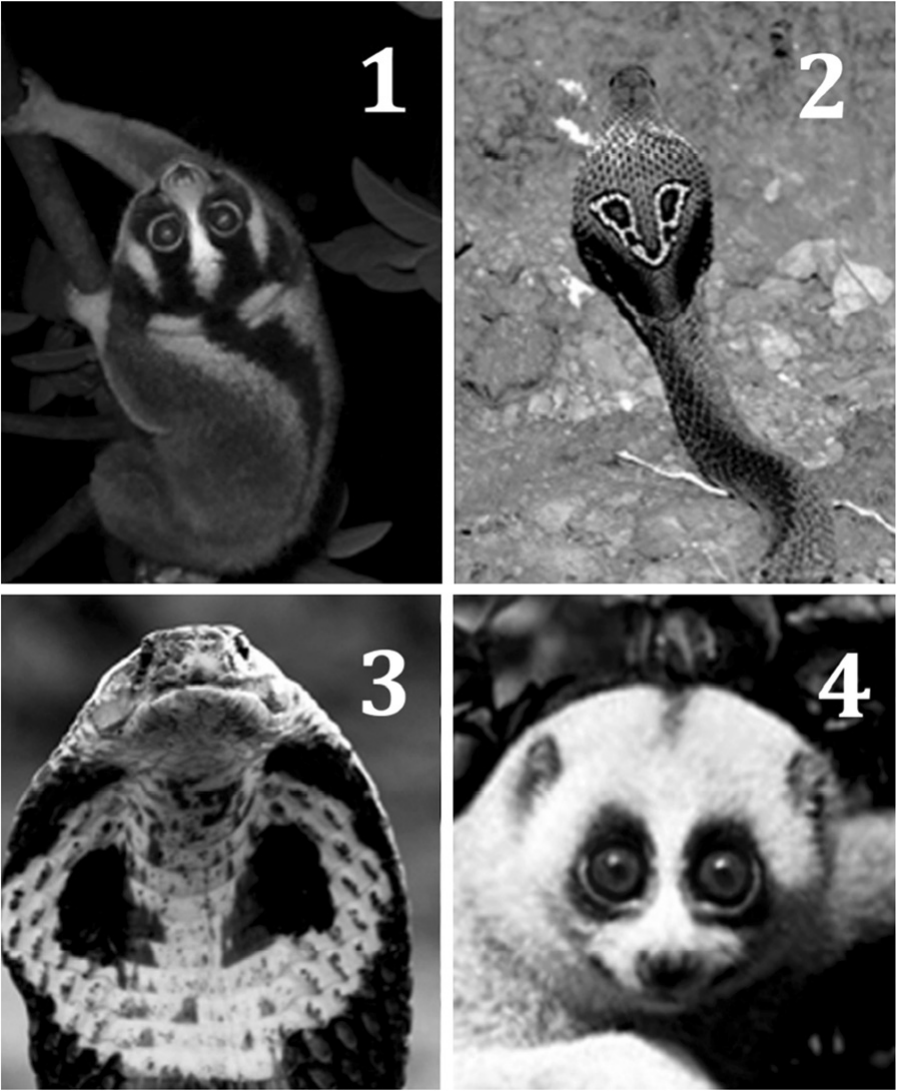
**Potential mimicry of spectacled cobras in Javan and Bengal slow lorises (1).** Javan slow loris (2) Spectacled cobra (rear view) (3) Spectacled cobra (front view) (4) Bengal slow loris.

Still’s [58] was the first anecdotal account of the uncanny resemblance of the slender loris (*Loris* sp*.*) to a cobra. Other authors have since remarked on lorises’ snake-like characteristics in regards to their defensive postures [59,60] and serpentine gait [61-63]. The distinctive expiratory pant-grunt produced during aggressive encounters by slow lorises (*Nycticebus*) [59,64,65] and slender lorises (*Loris*) [58,60] resembles remarkably the raspy hiss of a cobra during threatening displays [66]. Furthermore, slow lorises display facial markings undeniably akin to the eyespots and accompanying stripes of the spectacled cobra (*Naja naja*) (Figure 6). The dark contrasting dorsal stripe of these two species also closely resembles the body of a snake, particularly when viewed from above. We suggest in its evolutionary past that *Nycticebus* gained an adaptive advantage through Müllerian mimicry of *Naja naja.* For Müllerian mimicry to be effective, it is crucial that the animal mimic is recognised by a predator (or dupe) as another unpalatable or noxious model it is imitating. The mimicking of another’s warning signals consequently reduces the threat of attack [67,68]. For the predator to recognise the animal as an unpalatable prey species, the predator must already be aware of the other species’ undesirable characteristics [67]. Accordingly, at some point in time in their shared evolutionary history, the ranges of the mimic, the model and the dupe must have overlapped [53,57,69]. For mimicry to occur in one species, but not in other closely related species, would indicate a specific ecological pressure was driving the selection of mimetic traits in only that species, which was absent in those closely related to it [53,68].

We postulate that the *Nycticebus* mimicry evolved during a period of co-existence with *Naja naja*, at a time when environmental pressures would have favoured its selection. The genus *Naja* appears to have originated and diversified in Africa, subsequently travelling into Eurasia and across to Asia around 16 million years ago (MYA) [70-72]. These dates roughly correspond to the occurrence of a continuous land bridge from Africa to Asia in the early Miocene [73]. The origin of *Naja naja* in Asia is estimated at approximately ten MYA, where it still persists today in India, Pakistan, Sri Lanka and Bangladesh [71,72]. The earliest fossil record of lorises in Asia dates to eight MYA [74].

Around the time of a potential selection event, the climate in the Southeast Asia underwent a number of dramatic fluctuations, largely altering the vegetation [74-77]. Coinciding with intermittent land bridge formations, a band of drier more seasonally adapted woodland ran from north of the Malay Peninsula down as far as Java replacing the more tropical forests [76]. This habitat alteration may have benefitted some animals in allowing an easier migration south through the more savannah-like landscape, but for others it acted as a species isolation barrier [78,79]. For *Nycticebus* this change in habitat to a more open savannah-like environment and a different array of predators may have provided the initial selection impetus towards mimicry.

Lorises are arboreal primates, but when no continuous canopy is available, will occasionally venture over ground [80]. Terrestrial travel increases predation risk and is normally only attempted when no other option is present [81,82]. The climatic changes during the Pleistocene and the associated succession in vegetation from tropical forest to a more open savannah grassland environment may have increased the need for early *Nycticebus* to travel over ground. Consequently, the change in predation pressure caused by this adaptive shift may have triggered the move towards mimicry, whereby an advantage from mimicking a predator like *Naja naja* was gained. For aerial predators in particular, with their vision hampered by long grass, glimpses of the unmistakable markings of a spectacled cobra meandering across the ground between trees may have been enough to deter or at least postpone their intended attack.

## Conclusion

The theoretical framework discussed in this paper provides some support to hypotheses regarding the biochemistry, ecological function and evolution of slow loris venom. Local knowledge, severe injuries to conspecifics and medical records of humans and lorises cumulatively point towards the fact that loris venom is indeed a biological reality and potentially dangerous to its receiver [83]. Such information could be valuable to slow loris conservation projects. Detailed information on the ecology, habitat use and phylogenetic relationships of slow lorises is still scarce, and future studies may help to shed light on this topic. A closer examination of slow loris predator–prey, host-parasite and intraspecific interactions is vital to unravelling the complex network of selection pressures that have influenced the slow loris phenotype we see today.

## Competing interests

The authors declare that there are no competing interests.

## Authors’ contributions

KAIN conceptualised the long-term study on loris venom, and wrote the main manuscript. BGF contributed to the writing and designed and performed lab analyses. EJR and RSM contributed to wild field work and to the writing of the manuscript. All authors read and approved the final manuscript.

## Authors’ information

KAIN has studied the behaviour and ecology of all known species of slow and slender lorises in nine of their range countries in the wild and captivity since the early 1990s, and is Professor of Primate Conservation at Oxford Brookes University, UK. RSM conducted his PhD research on slow loris ecology and evolution, and is currently the research director of International Animal Rescue’s slow loris rescue centre in Indonesia. EJR conducted her PhD research on the ecological function of slow loris venom in Java. BGF has pioneered research in the venom composition of neglected groups, through using ecological, evolutionary, and functional genomics approaches to understand the evolution of venom systems. His novel work has led to redefining what constitutes venom, and thus its implications for drug design and development.

## Supplementary Material

Additional file 1 Video showing the components of the slow loris venom system, and an adult male anointing himself with venom during a conspecific battle for a female. Click here for file
